# Mangrove Facies Drives Resistance and Resilience of Sediment Microbes Exposed to Anthropic Disturbance

**DOI:** 10.3389/fmicb.2018.03337

**Published:** 2019-01-15

**Authors:** Cécile Capdeville, Thomas Pommier, Jonathan Gervaix, François Fromard, Jean-Luc Rols, Joséphine Leflaive

**Affiliations:** ^1^EcoLab, CNRS, INPT, UPS, Université de Toulouse, Toulouse, France; ^2^Ecologie Microbienne, INRA, UMR 1418, CNRS, UMR 5557, Université Lyon 1, Villeurbanne, France

**Keywords:** mangrove ecosystem, anthropic disturbance, wastewater discharge, *in situ* long term monitoring, microbial community, N-cycle

## Abstract

Mangrove forests are coastal ecosystems continuously affected by various environmental stresses and organized along constraint gradients perpendicular to the coastline. The aim of this study was to evaluate the resistance and resilience of sediment microbial communities in contrasted vegetation facies, during and after exposure to an anthropic disturbance. Our hypothesis was that microbial communities should be the most stable in the facies where the consequences of the anthropic disturbance are the most similar to those of natural disturbances. To test this, we focused on communities involved in N-cycle. We used an *in situ* experimental system set up in Mayotte Island where 2 zones dominated by different mangrove trees are daily exposed since 2008 to pretreated domestic wastewater (PW) discharges. These freshwater and nutrients inputs should increase microbial activities and hence the anoxia of sediments. We monitored during 1 year the long-term impact of this disturbance, its short-term impact and the resilience of microbial communities on plots where PW discharges were interrupted. Microorganism densities were estimated by qPCR, the nitrification (NEA) and denitrification (DEA) enzyme activities were evaluated by potential activity measurements and pigment analyses were performed to assess the composition of microbial photosynthetic communities. At long-term PW discharges significantly modified the structure of phototrophic communities and increased the total density of bacteria, the density of denitrifying bacteria and DEA. Similar effects were observed at short-term, notably in the facies dominated by *Ceriops tagal*. The results showed a partial resilience of microbial communities. This resilience was faster in the facies dominated by *Rhizophora mucronata*, which is more subjected to tides and sediment anoxia. The higher stability of microbial communities in this facies confirms our hypothesis. Such information should be taken into account in mangrove utilization and conservation policies.

## Introduction

Mangrove forests are coastal ecosystems of tropical and subtropical areas, continuously under tidal influence (Blasco, 1991; Spalding et al., 1997) and submitted to environmental constraints varying according to the seasons and to spatial gradients (salinity gradients, tidal cycle, sediment anoxia, soil instability) (Feller et al., 2010). These overall nutrient-poor forests (Boto and Wellington, 1984) are highly productive systems (Alongi, 2002). They provide varied food and material resources to human society and marine communities (Giri et al., 2011; Lee et al., 2014) and they are the main source of organic matter for the coastal marine food webs (Komiyama et al., 2000; Mohamed et al., 2016). They have also a high potential of carbon sequestration and storage due to rapid rates of net primary production and sedimentation (Komiyama et al., 2008; Donato et al., 2011; Alongi, 2012).

Microbial communities in mangroves sediments have a high diversity level (Andreote et al., 2012). These microorganisms allow the degradation of organic matter from vegetative materials (Holguin et al., 2001) and play a crucial role in nutrient cycles, particularly for nitrogen and phosphorous (Alvarenga et al., 2015). They contribute to the removal of nutrients like the nitrogen, and organic matter from sediments (Wu et al., 2008; Tam et al., 2009). The microorganisms involved in nitrogen cycle in mangrove ecosystem contribute through different metabolic processes: nitrogen (N_2_) fixation, nitrification, denitrification, ammonification, anaerobic ammonium oxidation (anammox) and dissimilatory nitrate reduction to ammonium (DNRA) (Alongi et al., 1992; Purvaja et al., 2008). These microorganisms participate in the degradation of organic nitrogen into inorganic nitrogen compounds (Alongi et al., 1992), available for the trophic chain, notably for the mangrove trees (Ganguly et al., 2009). Nitrogen can be removed from the sediments under the gaseous forms (N_2_, NO, N_2_O) by the denitrification and anammox processes carried out by denitrifying bacteria and anammox bacteria, respectively (Levy-Booth et al., 2014). The release of N_2_ from mangrove sediments seems to be more important by denitrification than anammox processes (Fernandes et al., 2012). Sediment surface also is partially covered with phototrophic and heterotrophic microorganisms embedded into a matrix of extracellular polymeric substances (Decho, 2000) which participate in the sediment stabilization against resuspension, plant growth promotion (Bouchez et al., 2013), and the chelation of toxic metals and other contaminants (Decho, 2000). Phototrophic microorganisms such as diatoms, green algae or cyanobacteria contribute to the primary production of the ecosystem and constitute a food source for heterotrophic protists and meiofauna (Bertrand et al., 2011).

As a whole, mangrove ecosystems are considered as very resilient to disturbances. The response of communities to disturbances and stresses depends on their resistance and resilience abilities, two components of ecosystem stability (Pimm, 1984). Resistance is the degree to which a community remains unchanged (Pimm, 1984), while the resilience can be defined as the rate of return to the initial state (Allison and Martiny, 2008). For both properties, either the structure of the community, or the associated functions can be considered. Microbial systems are usually considered as very resistant and resilient, because of the specific properties of microorganisms. Indeed, resistance is favored by their high metabolic flexibility, good physiological tolerance to environmental changes and short generation time. Resilience should be facilitated by their high abundances, high growth rates, high dispersal rate and high diversity (Fenchel and Finlay, 2004). Recent experimental studies emphasized contrasted abilities of microbial communities to recover from a similar disturbance (salinity stress) with either both structural and functional full recovery (Berga et al., 2017), only functional recovery (De Vrieze et al., 2017) or low recovery, in the case of extreme events (Hu et al., 2018).

In mangrove sediments, in addition to natural disturbances, microorganisms are directly exposed to anthropogenic pollutants that accumulate in sediments, such as organic contaminants (Zhang et al., 2014), oil spills (Muangchinda et al., 2013), domestic or industrial wastewaters (Tam, 1998). Structural modifications of the microbial communities can result from these anthropogenic inputs whether they are diffuse (Cao et al., 2011; Fernandes et al., 2014; Chakraborty et al., 2015) or controlled, as nutrient input (Li and Gu, 2013) or wastewater discharge (Tam, 1998).

Although the short-term response of microbial communities in mangrove sediments subjected to anthropogenic inputs is relatively well described, their actual resilience capacities are far less documented. Mangroves are very structured systems, organized in zones parallel to the coastline, along environmental gradients (salinity, nutrient availability, temperature), according to the sediment characteristics and the length of immersion by tides (Robertson and Alongi, 1992; Ball, 1998). Each zone is dominated by a few mangrove trees species adapted to the characteristic environmental conditions and associated with specific microbial communities in the sediments (Tam, 1998; Li and Gu, 2013; Wang et al., 2014). Though mangroves are often considered as a whole, these variations in environmental conditions and microbial community structure are associated with variations in the resistance and resilience properties of these communities. The aim of this study was to assess both resistance and resilience capacities of microbial communities in mangrove sediments exposed to anthropogenic disturbances. Our hypothesis was that the mangrove zone where the consequences of the applied disturbance are the most similar to those of natural disturbances should be the more stable.

To test this hypothesis, two contrasted mangrove zones were studied, respectively dominated by *Rhizophora mucronata* trees and *Ceriops tagal* trees. In the *Rhizophora*-dominated zone, closer to the seafront, the conditions are more constant, buffered by the tides but the longer immersion duration leads to longer anoxia of the sediment. In the *Ceriops*-dominated zone, the influence of tides is less important, the anoxia periods are shorter but the organisms are more submitted to aridity and salinity variations (Spalding et al., 2010; Tomlinson, 2016). The disturbance applied in these two mangrove zones was daily discharges of pretreated domestic wastewaters (PW). This input of nutrients and organic matter should increase microbial activity and consequently increase the anoxia of sediments. Based on *a priori* knowledge of the studied mangrove facies, including tide immersion time records, this disturbance should have a stronger similarity with the natural disturbances in the *R. mucronata*-dominated zone than in the *C. tagal* – dominated zone. The *in situ* experimental system used was set up in a mangrove of Mayotte Island in 2008 by Herteman et al. (2011), in order to assess the use of mangroves for bioepuration. It helped to evaluate the impact of PW on microbial communities after 1 year (Bouchez et al., 2013) and 4.5–5 years (Capdeville et al., unpublished) of discharges. In the present study, this experimental system was modified to assess both short- (after 3 weeks, 3, 8, and 12 months) and long-term (after 7.5–8.5 years) responses of microbial communities exposed to PW discharges, and their resilience after 12 months of the disruption of PW discharges.

## Materials and Methods

### Study Site

**FIGURE 1:**
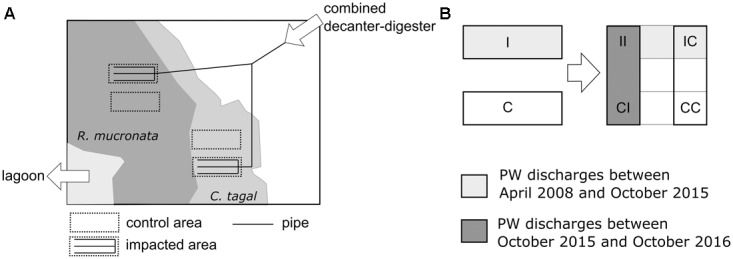
Schematic representation of the study site, with the impacted and control areas and the pipe network **(A)**, and of the modifications of the areas (I, impacted; C, control; II, impacted – still impacted; CC, control – still control; CI, control – then impacted; IC, impacted – then control) **(B)**.

**FIGURE 2:**
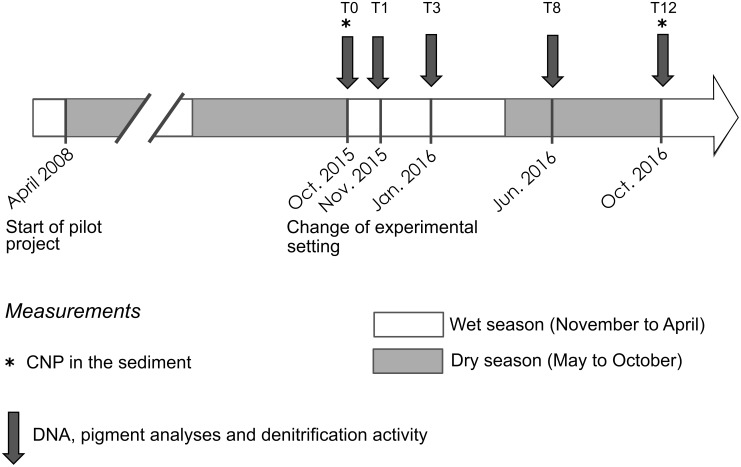
Organization of sampling during the study period from October 2015 to October 2016. Sampling time are designed by the number of months after T0 (from 1 to 12).

The *in situ* experimental system is localized in the mangrove of Chirongui Bay, South-West of Mayotte Island, a French department located in the Mozambique Channel, South-West of Indian Ocean (12°55′S, 45°09′E). Since April 2008, domestic wastewaters are continuously collected from Malamani village (250 inhabitants-equivalent), as described by Herteman et al. (2011). The wastewaters are pretreated in a horizontal primary settlement tank with integrated sludge digester. Then, they are carried through a pipe network to the mangrove areas. The areas of the *R. mucronata* mangrove zone are closer from the lagoon (about 400 m) than the ones in *C. tagal* mangrove zone (about 500 m), the latter being less subjected to tides (in mean 0.88 h/day of immersion against 4.33 h/day for the first zone). The PW were mainly composed of organic matter, nitrogen (mainly ammonium form) and phosphorus nutrients, discharged in the mangrove areas with surface loading rates (for dry season, in g.m^-2^.d^-1^) of 6.1 for chemical oxygen demand, 2.0 for biological oxygen demand after 5 days, 2.13 for suspended solids, 0.99 for total nitrogen and 0.111 for total phosphorus (data obtained for 2015–2017 from SIEAM (Syndicat des Eaux et d’Assainissement de Mayotte, Mayotte, France). From April 2008 to October 2015, PW have been discharged at a volume of 10 m^3^ once per day, during a low tide, for 1 h onto mangrove areas (around 675 m^2^, 15 m × 45 m) respectively dominated by the mangrove trees *Rhizophora mucronata* Lam and *Ceriops tagal* (Perr.) C. B. Robinson (Figure 1A), giving a hydraulic loading rate of 14.8 L.m^-2^.d^-1^, which is equivalent to a rainfall event of 14.8 mm. Near these two “impacted” areas, two other areas not subjected to PW discharge were used as “control” areas (Figure 1A). In October 2015 (at time T0), the experimental setting was changed to assess the resilience and the resistance capacities of microbial communities for 12 months (Figure 1B). In each mangrove zone, four different areas were delimited (around 225 m^2^, 15 m × 15 m): one part of the initial impacted area was still impacted in the new discharge network (C-II and R-II areas in *C. tagal* and *R. mucronata* mangrove zones, respectively) while in the another one the PW discharge were stopped at the T0 (C-IC and R-IC areas), one part of the initial control area was still a control after the T0 (C-CC or R-CC areas) while the another one received PW discharge since the T0 (C-CI or R-CI areas). In each area, 4 plots (1 m^2^) were randomly chosen, marked out and used for all the samplings. The short-term impact of PW supply and the resilience capacities of microbial compartment were evaluated at T0, T0 + 3 weeks, T0 + 3 months, T0 + 8 months and T0 + 12 months, during the dry season (May–October) and the wet season (November–April) (Figure 2).

### Sediments and Porewater Composition

The salinity and temperature of water were measured directly in the field in residual pools (surface water) and in piezometers (deep water, approximately 1 m) at low tide and samples were taken and stored at 4°C for later determination of nutrient (NO_3_^-^, NO_2_^-^, NH_4_^+^ and PO_4_^3-^) concentrations. Analyses were performed by the ARVAM Laboratory (La Réunion, France) using classic colorimetric methods according to standard methods (American Public Health Association [APHA] et al., 1992). Mean values were obtained from 2-day measurements under similar hydrological conditions, in the upper and lower parts of the areas (total of 4 measures).

In each area, one sediment core (1 cm-depth) per plot was carried out with a syringe (50 mL). The sediment samples were dried and weighed after desiccation in an air oven at 105°C during 2 days. N and C quantities were measured in a powder of dry sediments with a Thermo Fisher Flash 2000 elemental analyzer (Thermo Fisher Scientific, United States). Total phosphorus (P) was measured in acid condition after a persulfate oxidation using the ammonium molybdate spectrophotometry method.

### Estimation of Microbial Densities

#### Sediment Sampling

In each area, one sediment core (10 cm-depth) per plot (4 cores in total) was carried out with a syringe (50 mL), placed into 350 mL of RNAlater solution, homogenized and maintained at 4°C until treatment of samples at the laboratory. RNAlater solution was used in order to protect RNA contents for other experiments. This solution was prepared only with RNAse-free glassware and water treated with dimethyl dicarbonate (DMDC). First, the DMDC-water was prepared with 1 mL of DMDC (Merck, Germany), 50 mL of ethanol absolute and ultrapure water QSP 1 L. This DMDC-water was incubated overnight at 37°C before autoclaving (at 121°C, 1 bar, and 20 min) and was used for all solutions. Then 1.5 L of RNAlater solution were made with 935 mL of DMDC-water, 700 g of ammonium sulfate (Sigma-Aldrich, France), 25 mL of 1 M sodium citrate solution (Sigma-Aldrich, France) and 40 mL of 0.5 M EDTA (Sigma-Aldrich, France). The pH of RNAlater solution was adjusted at 5.2.

#### Quantitative PCR on 16S *r*DNA, *nos*Z Clade I and *amo*A Genes

The total density of bacteria in sediments was determined with the abundance of the bacterial 16S *r*DNA gene. Standard curves and positive controls were obtained using serial dilution of DNA extracted from an *Escherichia coli* culture. The densities of bacteria involved in functional processes were determined with the *nos*Z clade I gene for denitrification and the *amo*A gene for nitrification. The latter was targeted also to determine the density of nitrifying archaea. Standards were amplified from mangrove samples in a total volume of 25 μL, containing 25 (*nos*Z clade I) or 10 (*amo*A) ng of DNA template, 1 U of GoTaq^®^ G2 Flexi DNA polymerase (Promega), 1X GoTaq^®^ G2 Flexi buffer, 1.5 mM of MgCl_2_, 0.8 mM dNTP, 1 μM (*nos*Z clade I) or 0.2 μM (*amo*A) of each primer and 0.3 (*nos*Z clade I) or 0.2 (*amo*A) mg.mL^-1^ of BSA. Thermal cycling conditions and primers used for each reaction are described in Table 1. Every PCR started with an initial step at 95°C for 10 min and finished by a final step at 72°C for 3 min. PCR fragments (259 bp for nosZ clade I, 491 bp for bacterial *amo*A, 635 bp for archaeal *amo*A) were next cloned into a pGEMT plasmid using the pGEM^®^-T Easy Vector System II (Promega) according to the manufacturer’s recommendations. Plasmids extractions were carried out with the NucleoSpin^®^ Plasmid kit (Macherey-Nagel, France) following the manufacturer’s instructions. Recombined plasmids were linearized with the *EcoR*I restriction enzyme (Promega). Standard curves and positive controls were obtained using serial dilution of linearized plasmids containing 10^1^ to 10^7^ gene copies.μL^-1^. Water was used as a negative control.

**Table 1:** PCR primers (Eurofin, Germany) and thermal conditions (AOB, ammonium oxidizing bacteria; AOA, ammonium oxidizing archaea).

Target	Gene	Method	Primers	Sequence (5′ – 3′)	Thermal conditions			No. of cycles	Type	Reference
Bacteria	16S	qPCR	Primer P1 Primer P2	CCTACGGGAGGCAGCAG ATTACCGCGGCTGCTGG	95°C, 15 s	61.5°C, 45 s	–	40	Classic	Muyzer et al., 1993
Denitrifying bacteria	*nos*Z clade I	PCR	nosZ1F nosZ2R	WCSYTGTTCMTCGACAGCCAG ATGTCGATCARCTGVKCRTTYTC	95°C, 15 s	67°C-62°C, 30 s	72°C, 30 s	6 and 35	Touch down	Henry et al., 2006
		qPCR	nosZ1F nosZ2R	WCSYTGTTCMTCGACAGCCAG ATGTCGATCARCTGVKCRTTYTC	95°C, 10 s	67°C-62°C, 45 s	–	6 and 40	Touch down	Henry et al., 2006
AOB	Bacterial *amo*A	PCR	amoA1F amoA2R	GGGGTTTCTACTGGTGGT CCCCTCKGSAAAGCCTTCTTC	95°C, 30 s	55°C, 30 s	72°C, 45 s	35	Classic	Rotthauwe et al., 1997
		qPCR	amoA1F amoA2R	GGGGTTTCTACTGGTGGT CCCCTCKGSAAAGCCTTCTTC	95°C, 15 s	58°C, 2 min	–	40	Classic	Rotthauwe et al., 1997
AOA	Archaeal *amo*A	PCR	amoA19F Crenamo A616r48x	ATGGTCTGGCTWAGACG GCCATCCABCKRTANGTCCA	95°C, 30 s	53°C, 1 min	72°C, 1 min	35	Classic	Leininger, personal communication, 2006; Tourna et al., 2008
		qPCR	amoA19F Crenamo A616r48x	ATGGTCTGGCTWAGACG GCCATCCABCKRTANGTCCA	95°C, 25 s	55°C, 2 min 45 s	–	40	Classic	Leininger, personal communication, 2006; Tourna et al., 2008


#### DNA Extraction

Before DNA extraction, some subsamples of the sediments were washed three times with PBS 1 X (AccuGENE, Lonza, Switzerland) to remove the RNAlater solution. DNA extraction was performed on 2.5 g of wet sediments after an RNA extraction with the RNA Powersoil^®^ DNA Elution Accessory kit (MoBio, Quiagen, United States) according to manufacturer’s instructions. The quality of total DNA was checked using a NanoDrop 1000 spectrophotometer (Thermofisher Scientific, United States) and by gel electrophoresis (agarose, 1% in TAE 0.5X).

Quantitative PCRs (qPCR) were performed in triplicate for each DNA extract. The quantification was based on the fluorescence intensity of the SYBR green dye, which binds to double-stranded DNA. The qPCR analyses of sediment samples were carried out in a final volume of 20 μL containing 1X of Takyon^TM^ Rox SYBR^®^ MasterMix dTTP Blue (Eurogentec, Belgium), 0.3 mg.mL^-1^ of BSA (Promega), 5 μL (16S rDNA and *amoA* genes) or 4 μL (*nosZ* clade I) of 1/100-DNA samples or standards and 0.3, 0.5, and 0.2 μM of each primers (Eurofin, Germany) for 16S rDNA, *nosZ* clade I, and *amoA* genes respectively (Table 2). qPCRs were carried out on the StepOnePlus Real-Time PCR System (Applied Biosystems, United States) starting by an initial enzyme activation at 95°C for 3 min for each qPCR. The thermal cycling conditions used for qPCR of each gene are described in Table 1. Serial dilutions of the DNA extracted from sediment samples were quantified and compared to check the presence of PCR inhibitors but no inhibition was detected. Melting curves were analyzed using StepOne^TM^ v2.3 software to confirm the specificity and efficiency of the amplification and the quantifications of gene in samples were deduced from standard curve. Results were expressed in number of gene copies per gram of sediment dry weight.

**Table 2:** Physical–chemistry of surface water and sediment porewater assessed at T0 in impacted and control areas (mean ± SE, *n* = 4 for *R. mucronata, n* = 2–4 for *C. tagal*).

			Temperature (°C)	Conductivity (mS cm^-1^)	Salinity (psu)	PO_4_^3-^ (μM)	NH_4_^+^ (μM)	NO_3_^-^ (μM)	NO_2_^-^ (μM)
Surface water	*C. tagal*	Control Impacted	23.2 ± 1.2 22.8 ± 0.2	61.3 ± 1.6 53.6 ± 2.7	41.1 ± 1.3 35.3 ± 2.0	0.83 ± 0.01 0.37 ± 0.15	37.97 ± 20.28 293.78 ± 147.20	1.05 ± 0.21 1.28 ± 0.64	0.61 ± 0.11 4.20 ± 0.08
	*R. mucronata*	Control	24.4 ± 0.1	57.8 ± 0.5	38.6 ± 0.3	0.75 ± 0.28	4.16 ± 3.78	0.18 ± 0.10	0.07 ± 0.01
		Impacted	24.8 ± 0.4	51.7 ± 2.5	34.0 ± 1.8^**∗**^	13.2 ± 11.17	671.10 ± 423.06^**∗**^	0.51 ± 0.10	0.50 ± 0.12^**∗**^
Deep water	*C. tagal*	Control	24.8 ± 0.2	59.1 ± 2.3	39.5 ± 1.7	5.93 ± 1.28	0.38 ± 0.23	0.35 ± 0.20	0.06 ± 0.01
		Impacted	25.0 ± 0.0	67.3 ± 0.3	45.8 ± 0.2	10.79 ± 2.10	0.14 ± 0.09	0.39 ± 0.22	0.07 ± 0.00
	*R. mucronata*	Control	24.8 ± 0.3	56.0 ± 0.9	36.5 ± 0.5	4.56 ± 2.12	0.20 ± 0.07	0.24 ± 0.03	0.08 ± 0.02
		Impacted	24.8 ± 0.0	61.8 ± 2.0^**∗**^	41.6 ± 1.5^**∗**^	11.79 ± 2.04^**∗**^	1.31 ± 0.41^**∗**^	0.16 ± 0.04	0.06 ± 0.00


*Asterisks indicate significant differences with the respective control (^∗^*p* < 0.05).*

RT-qPCR were supposed to be made on the same samples, reason why RNAlater was used. However, the quality of RNA was not sufficient to allow these analyses.

### Estimations of Potential Nitrification (NEA) and Denitrification (DEA) Enzyme Activities

NEA and DEA were performed on sediment samples collected during the 5 sampling campaigns. In each area, one sediment core (10 cm-depth) on each of the 4 plots was carried out with a syringe (50 mL), homogenized into 100 mL-tube, kept in a cooler box during transport and stored at 4°C until treatment of sediment samples few days later at the laboratory to limit the modification of denitrification activity.

Potential nitrification activity (PNA) was measured using a modified method described by Dassonville et al. (2011). Briefly, samples of fresh soil (3 g dry weight equivalent) were incubated for 10 h with 30 mL of (NH_4_)_2_SO_4_ (1.25 mg N.L^-1^) using continuous shaking (140 rpm, 28°C). To simulate marine environment each sample was submerged with a saline solution (30g NaCl.L^-1^). Subsamples (1 mL) were collected at 2, 4, 6, 8, and 10 h, filtered (0.20 μm pore size) and stored at -20°C. The NO_3_^-^ and NO_2_^-^ concentrations were analyzed using a colorimetric assessment using a SmartChem 200 (AMS Alliance).

For the measure of DEA, 2 flasks (150 mL) were prepared for each sample and filled with 10 mL of sediments collected with a syringe (10 mL). All flasks containing sediments were pre-incubated at 30°C during 24 h to re-activate bacteria. After 24 h, 50 mL of incubation medium were added to each flask. This medium was composed by deoxygenated demineralized water containing N-NO_3_ (100 mg.L^-1^) and organic C (glucose) (50 mg.L^-1^), at a salinity of 30 psu. Then the flasks were sealed with rubber stoppers and deoxygenized by diffusion of N_2_ during 20 min. Then, 15 mL of incubation medium saturated with acetylene were added in one flask per sample, the other one receiving medium without acetylene (negative control). All the flasks were incubated in the dark at 30°C with agitation. After 3 and 6 h of incubation, the flasks were vigorously stirred for 20 s and 6 mL of gas were sampled and injected in vacuum 8 mL-vials. Then, the 6 mL of sampled gas were replaced by 6 mL of N_2_. The acetylene blocks the last step of denitrification (formation of N_2_ from N_2_O), allowing the accumulation of N_2_O in the flasks (Sorensen, 1978). Finally, N_2_O concentrations of all gas samples were analyzed on a gas chromatograph (VARIAN 3800) equipped with a 63Ni capture detector. The carrier gas was a mixture of argon/methane (90/10 v/v). The separation was made on a Porapak Q column at 80°C, the injector and detector temperatures were 120 and 280°C, respectively.

After the incubations, the ash-free dry mass (AFDM) of each sediment sample was determined after combustion (at 550°C, for 8 h).

### Assessment of the Structure of Phototrophic Microbial Communities

#### Extraction of Photosynthetic Pigments in Sediments

Two sediment samples (1 cm-depth) were collected in each plot of all the areas with a syringe (50 mL), kept in a cooler box during transport, and stored at -20°C until storage at -80°C at the laboratory. Before extraction, sediment samples were freeze-dried and homogenized. The pigments from 0.5 g of dry sediments were extracted with 5 mL of methanol buffered with 2% of 1 M-ammonium acetate (Sigma-Aldrich, France). After 2 min in an ultrasound cold bath and at maximum power, samples were kept in the dark at -20°C for 15 min before centrifugation (High Conic Rotor, Thermo Scientific, 3220 *g*, 2°C, and 5 min). Supernatants were collected and the pellets were re-extracted as described above. The pooled supernatants were filtered on 0.2 μm PTFE membrane syringe filter (Ø 13 mm, VWR International, United States) and stored a few days at -80°C before High Performance Liquid Chromatography (HPLC) analysis. To prevent degradation of pigments, extractions were performed under dark conditions and samples stored on ice during handling.

#### HPLC Analyses

High Performance Liquid Chromatography analyses were performed with a 100 μL-loop auto-sampler and a quaternary solvent delivery system coupled to a diode array spectrophotometer (LC1200 series, Agilent Technologies, United States). The mobile phase was prepared [solvent A: 70:30 (v/v) methanol:1 M ammonium acetate and solvent B: 100% methanol] and programmed (minutes; % solvent A; % solvent B): (0; 75; 25), (1; 50; 50), (20; 30; 70), (25; 0; l00), (32; 0; 100) according to the analytical gradient protocol described by Barlow et al. (1997). The column was then reconditioned to original conditions over a further 12 min. Pigment separation was performed through a C8, 3 μm-column (MOS-2 HYPERSIL, Thermo Scientific, United States). The diode array detector was set at 440 nm to detect carotenoids, at 665 nm for chlorophylls and pheopigments. Pigments were identified by comparing their retention time and absorption spectra with those of pure standard pigments (DHI LAB products, Denmark). Each pigment concentration was calculated by relating the peak area of its chromatogram with the corresponding area of calibrated standard using ChemStation software (version A.10.02, Agilent technologies). For each sample, we worked on the ratios between total pigment concentrations and chlorophyll *a* concentration. From this matrix, we calculated similarities with the Bray Curtis index between all areas of *C. tagal* and *R. mucronata* mangrove zones at each sampling time.

### Statistical Analyses

Statistical analysis were performed using the PAST software (Paleontological Statistics, versions 2.17 and 3.06) (Hammer et al., 2001). The normality was checked on each dataset with the Shapiro–Wilk test and data were transformed if needed. When data were normally distributed, two-way ANOVAs were used to test the effects of mangrove zone (*R. mucronata* vs. *C. tagal*) and treatment (control vs. impacted areas), followed by a Tukey *post hoc* test. The non-parametric Kruskal–Wallis test was used with Mann–Whitney test for pairwise comparisons of non-parametric data. Statistical differences between sampling campaigns (T0, T0 + 3 weeks and T0 + 3, 8, and 12 months) were analyzed with one-way ANOVA followed by Tukey *post hoc* test or by the non-parametric Kruskal–Wallis test with Mann–Whitney test for pairwise comparisons according to the type of distribution. Data given in the text are means ± SE. For all statistical analyses, significance was inferred at *p* < 0.05 (noted ^∗^ if *p* ≤ 0.05, ^∗∗^*p* ≤ 0.01, ^∗∗∗^*p* ≤ 0.001).

## Results

### Sediment and Porewater Composition

The temperature, conductivity, salinity and nutrient concentrations were measured at T0 in surface water and porewater (within piezometers) of sediments. The results are given in Table 2. Because of a lack of water in some *C. tagal* sampling points, only 2 replicates are available for this mangrove zone, so that the statistical analyses have been performed only for the *R. mucronata* zone (4 replicates). In surface water, the PW discharges induced a decrease of the salinity, and an increase in NH_4_^+^ and NO_2_^-^ concentrations. The observations were similar for the *C. tagal* zone. In porewater, an increase of salinity and conductivity in *R. mucronata* impacted zone was associated with an increase of NH_4_^+^ and PO_4_^3-^ concentrations. The tendency was similar in the *C. tagal* zone, except for the NH_4_^+^ concentration, lower in the impacted area.

**FIGURE 3:**
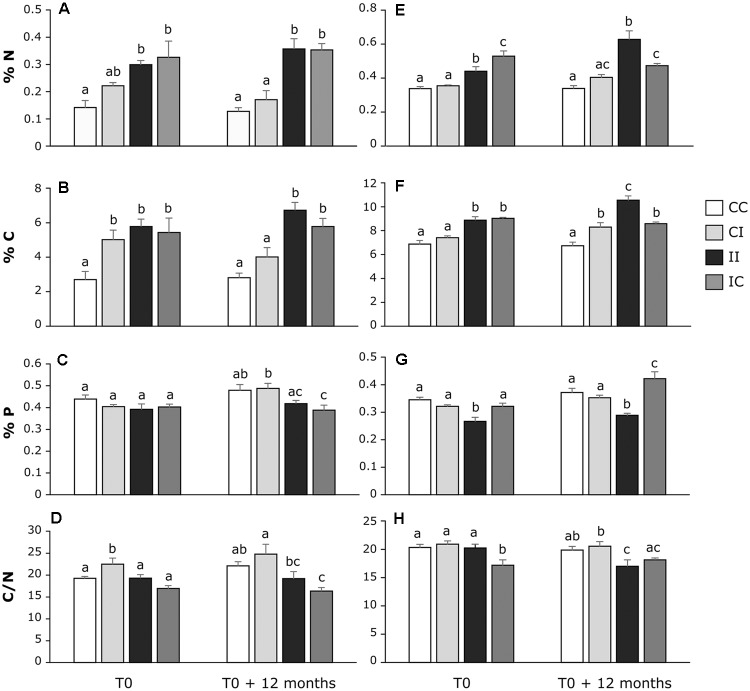
Dynamic of N **(A,E)**, C **(B,F)** and P **(C,G)** contents (%) and C:N **(D,H)** ratio in the first sediment layer (0–1 cm) of each area in the *C. tagal* **(A–D)** and *R. mucronata* **(E–G)** mangrove zones, at T0 and T0 + 12 months. Letters indicate statistically homogeneous groups (*post hoc* test after ANOVA or Kruskal–Wallis) (mean ± SE, *n* = 4).

The sediment elementary composition was assessed at the sediment surface in each area of *C. tagal* and *R. mucronata* mangrove zones at T0 and after 12 months (Figure 3). Overall, the elementary composition was different between the two mangrove zones, with higher N (0.44% ± 0.02) and C (8.29% ± 0.23) contents, and lower P (0.34% ± 0.01) content in the *R. mucronata* zone than in the *C. tagal* zone (0.25% ± 0.02, 4.78% ± 0.30, 0.43% ± 0.01 respectively). Until T0, the CC and CI areas, and the II and IC areas had received the same treatment, i.e., no PW discharge and daily PW discharges, respectively. Nevertheless, some significant differences in the elementary composition of the sediment in similar areas were observable, notably in the *C. tagal* mangrove zone with higher N and C contents in the CI area than in the CC area. In this zone, the total C, N, and P contents and C:N ratio of the sediments were rather stable during the experiment (Figures 3A–D) in CC and II areas. The PW discharges significantly increased the N and P contents, while they had no effects on P content and C:N ratio. The modifications of the treatment in CI and IC areas did not modify the elementary composition of the sediment after 12 months. In the *R. mucronata* mangrove zone, while the composition of the sediment in the CC area was stable over time, it varied in the II area, with an increase in C (*p* = 0.021) content and a trend to increase in N (*p* = 0.056) content (Figures 3E,F). As for the other mangrove zone, PW discharges induced an increase in N and C contents. It also induced a decrease in P content and a small decrease in C:N ratio (Figures 3E–H). Twelve months after the end of PW discharges, N and P contents were significantly lower in IC area than in II area. During the same period, PW discharge induced an increase in C content in CI area compared to CC area.

### Microbial Communities

#### Total Density of Bacteria

**FIGURE 4:**
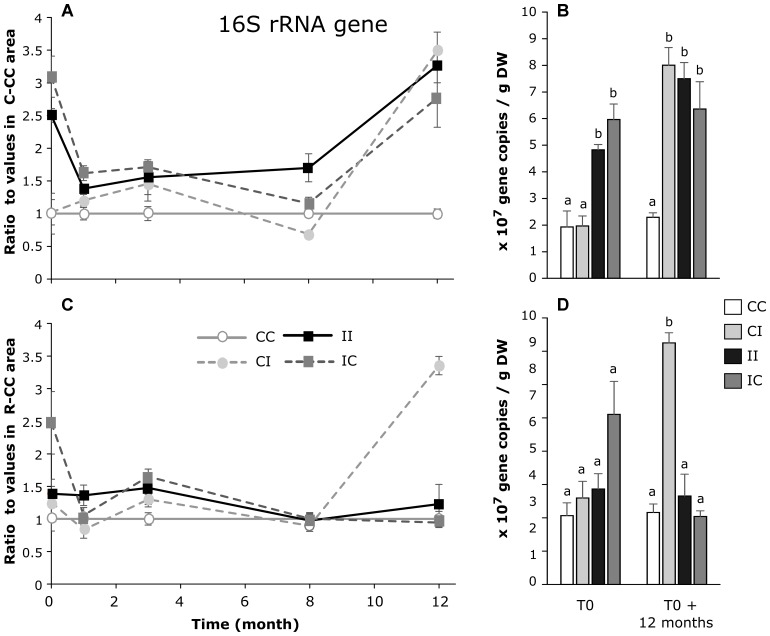
Dynamic of the ratios of 16S *r*DNA gene copies/g DW (dry weight) between sediments from each area and from the CC area of *C. tagal* **(A)** and *R. mucronata* **(C)**. The numbers of gene copies/g DW are given for T0 and T0 + 12 months **(B,D)**. Letters indicate statistically homogeneous groups (*post hoc* test after Kruskal–Wallis) (mean ± SE, *n* = 4).

The total density of bacteria was followed through the number of copies of 16S *r*DNA gene in all areas of *C. tagal* and *R. mucronata* mangrove zones during 12 months (Figure 4). The total density of bacteria was very variable overtime in the controls, and depended on the season. Therefore, in order to observe the effects of treatments, the data were normalized by the mean value of the respective control, for each mangrove zone and for each sampling time (Figures 4A,C). In the *C. tagal* mangrove zone, at all the sampling times, the number of 16S *r*DNA gene copies was significantly higher in C-II impacted area (5.39 × 10^7^ ± 3.23 × 10^6^ 16S *r*DNA copies/g DW) compared to the C-CC control area (1.95 × 10^7^ ± 3.46 × 10^6^ 16S *r*DNA copies/g DW) (Figure 4B). Twelve months after the PW discharges were stopped, the density of bacteria in the C-IC area was still similar to the one in the C-II area. In contrast, during the same duration, the density of bacteria in the C-CI area reached the one of C-II area. In the *R. mucronata* mangrove zone, there was no effect of the PW discharges on the bacterial density at the beginning of the experiment, but after 12 months, a significant increase was observable in the R-CI area (Figures 4C,D).

#### Density of Functional Groups

The abundances of denitrifying bacteria and nitrifying bacteria and archaea were followed overtime by the number of copies of *nos*Z clade I gene and the *amo*A gene, respectively. For the three genes, the number of gene copies was strongly variable overtime with maximum values for all areas at 8 months, like for the 16S gene. On the whole, the functional community was dominated by the denitrifying bacteria, from 2.7 to 6.6 times (for C-CC and C-CI areas, respectively) more abundant than the nitrifying archaea, which were 3.5-4.7 times (for C-IC and C-CC areas) more abundant than the nitrifying bacteria. For the *R. mucronata* mangrove zone, the denitrifying bacteria were 2.0–4.7 times (for R-CC and R-II areas) more abundant than the nitrifying archaea, which were 4.6–7.0 times (for R-CI and R-CC areas) more abundant than the nitrifying bacteria. These values were very variable over time notably in the control areas, depending on the season. To make comparisons easier, we focused on gene densities at the same period of the year, in October 2015 and October 2016.

**FIGURE 5:**
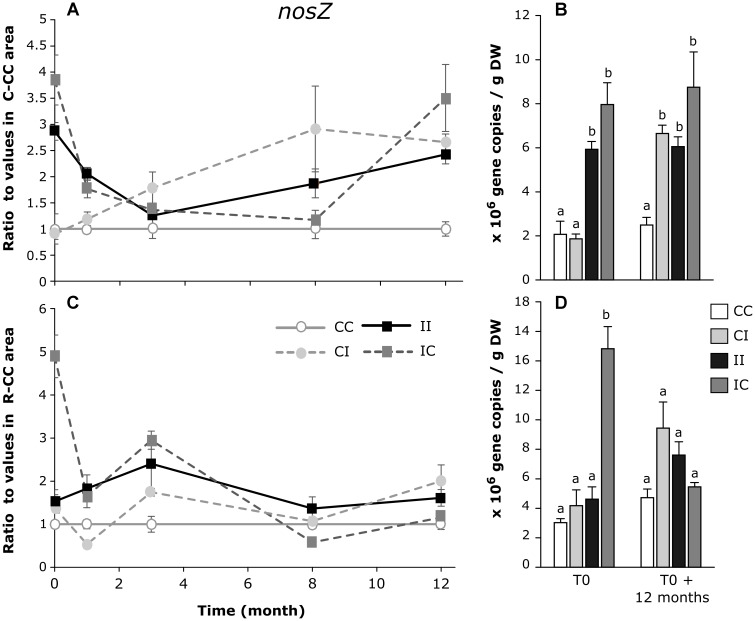
Dynamic of the ratios of *nosZ* clade gene copies/g DW (dry weight) between sediments from each area and from the CC area of *C. tagal* **(A)** and *R. mucronata* **(C)**. The numbers of gene copies/g DW are given for T0 and T0 + 12 months **(B,D)**. Letters indicate statistically homogeneous groups (*post hoc* test after Kruskal–Wallis) (mean ± SE, *n* = 4).

In the *C. tagal* mangrove zone, the density of denitrifying bacteria followed the same pattern than the density of total bacteria: the PW discharges induced an increase in the number of *nosZ* clade I copies and during the 12 month-experiment this number was stable in the C-IC area and increased in the C-CI area (Figures 5A,B). The effect of PW discharges on denitrifying bacteria was less clear in the *R. mucronata* mangrove zone, with higher values at T0 in the R-IC area, and similar values in all the areas after 12 months (Figures 5C,D).

**FIGURE 6:**
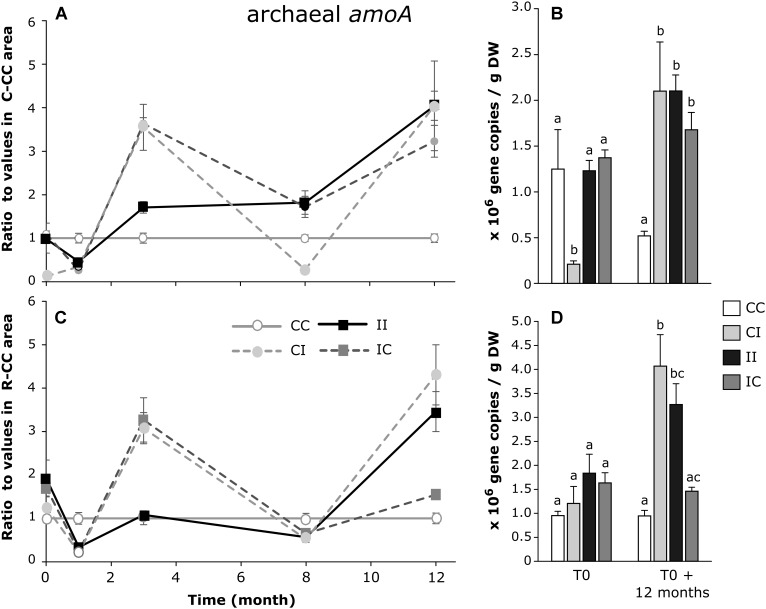
Dynamic of the ratios of archeal *amoA* gene copies/g DW (dry weight) between sediments from each area and from the CC area of *C. tagal* **(A)** and *R. mucronata* **(C)**. The numbers of gene copies/g DW are given for T0 and T0 + 12 months **(B,D)**. Letters indicate statistically homogeneous groups (*post hoc* test after Kruskal–Wallis) (mean ± SE, *n* = 4).

As shown in Figure 6, the effects of PW discharges on the number of archaeal *amo*A gene copies were not very clear. At T0, there was a lower value only in the C-CI area, and no differences in the *R. mucronata* zone. After 12 months, all the values were higher than the control area, in the *C. tagal* zone (Figure 6B) while in the *R. mucronata* zone, the gene density increased in the R-CI and R-II areas.

**FIGURE 7:**
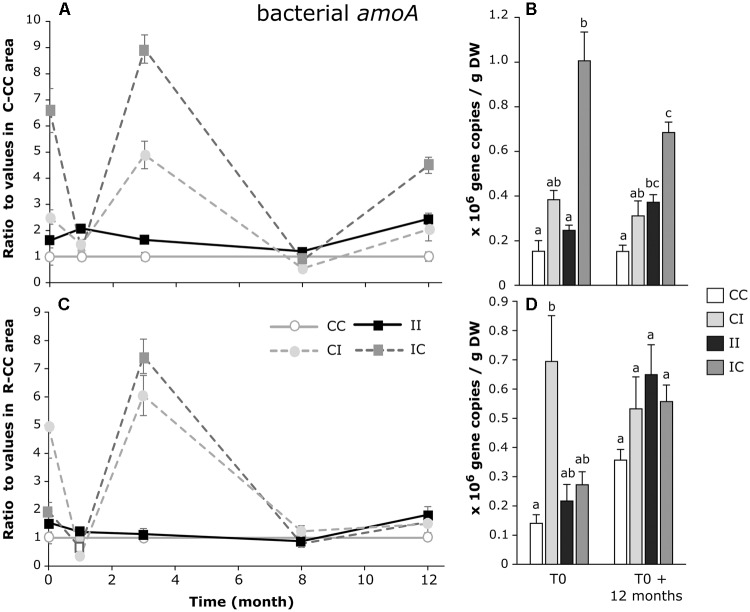
Dynamic of the ratios of bacterial *amoA* gene copies/g DW (dry weight) between sediments from each area and from the CC area of *C. tagal* **(A)** and *R. mucronata* **(C)**. The numbers of gene copies/g DW are given for T0 and T0 + 12 months **(B,D)**. Letters indicate statistically homogeneous groups (*post hoc* test after Kruskal–Wallis) (mean ± SE, *n* = 4).

The number of bacterial *amo*A gene copies strongly varied over time, notably in the areas where the treatment was modified at T0 (IC and CI) (Figures 7A,C). In the *C. tagal* zone, the effect of PW discharges was variable between areas that had received the same treatment, with an increase compared to control in C-IC and no effect in C-II (Figure 7B). Few modifications occurred after 12 months. In the *R. mucronata* zone, there was no clear effect of the PW discharges (Figure 7D).

#### Potential Nitrification Activity in Relation to Ammonium Oxidative Bacteria

**FIGURE 8:**
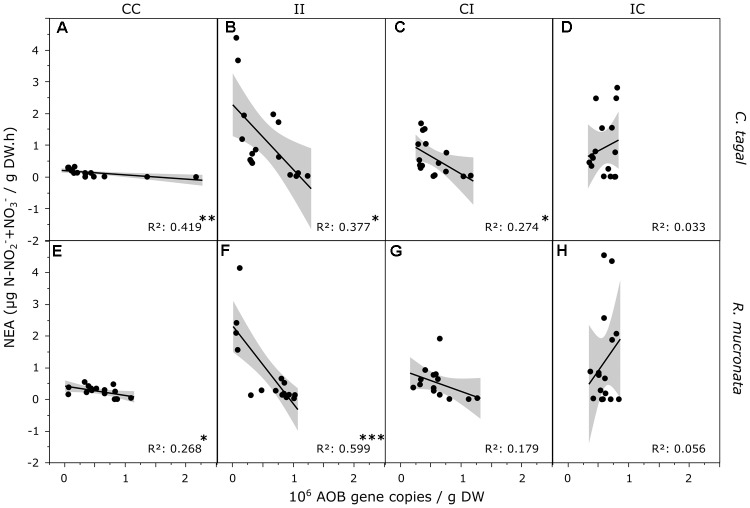
Correlations between AOB densities and NEA in *C. tagal* **(A–D)** and *R. mucronata* **(E,F)** mangrove zones, in the CC **(A,E)**, II **(B,F)**, CI **(C,G)**, IC **(D,H)** areas. Data from T1 to T12 were included. Asterisks indicate significant correlations (^∗^*p* < 0.05, ^∗∗^*p* < 0.01, ^∗∗∗^*p* < 0.001).

The potential nitrification activity (NEA) carried out by AOA, AOB and nitrite oxidizing bacteria was assessed by measuring NO_2_^-^/NO_3_^-^ net production in sediments from all areas of *C. tagal* and *R. mucronata* mangrove zones. While the correlation between NEA and AOA variation did not show any particular trend regarding both zones and all treatments (data not shown), the correlation between NEA and AOB was strongly and negatively impacted in the CI, and even more importantly in the II treatment (Figure 8). In contrast, this negative influence was released in the IC treatment, similar to what was observed in the controls, suggesting a potential resilience of this relation in time. Similar trends were observed for the relationship between NEA and AOA + AOB.

#### Potential Denitrification Activity

**FIGURE 9:**
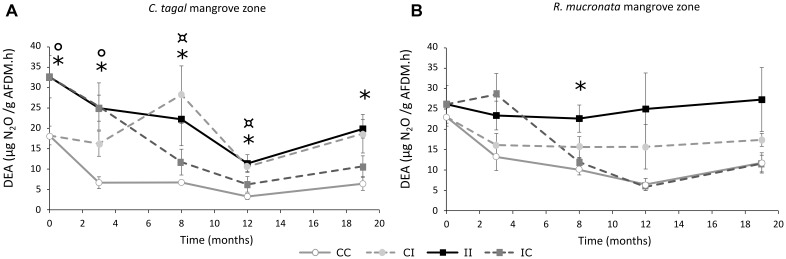
Dynamic of potential denitrification activity in sediments of *C. tagal* **(A)** and *R. mucronata* **(B)** for all areas. Significant differences (*post hoc* test after ANOVA or Kruskal–Wallis) are indicated with ^∗^*p* ≤ 0.05 for differences between control (CC) and impacted areas (II), ¤*p* ≤ 0.05 for differences between control and new impacted areas (CI) and °*p* ≤ 0.05 for differences between control and new control areas (IC) (mean ± SE, *n* = 4).

The potential denitrification activity (DEA) carried out by the denitrifying bacteria was assessed by measuring N_2_O production in sediments from all areas of *C. tagal* and *R. mucronata* mangrove zones. In the *C. tagal* mangrove zone, the DEA was significantly higher in the impacted area than in the control area, at all the sampling dates (Figure 9A). In the *R. mucronata* mangrove zone, the DEA followed the same trend although the differences were not significant (Figure 9B). A decrease of N_2_O production was observed in C-IC and R-IC areas after 8 months without PW discharge. It was more marked in the *R. mucronata* mangrove zone. In contrast, an increase of N_2_O production was observed in C-CI, corresponding to the short-term impact of PW after 8 months of discharge (Figure 9). The DEA became similar in C-II and in C-CI areas. A similar trend was observed in *R. mucronata* mangrove zone but the N_2_O production in R-CI area did not reach the values in R-II impacted area (Figure 9).

### Structure of Microbial Photosynthetic Communities

**FIGURE 10:**
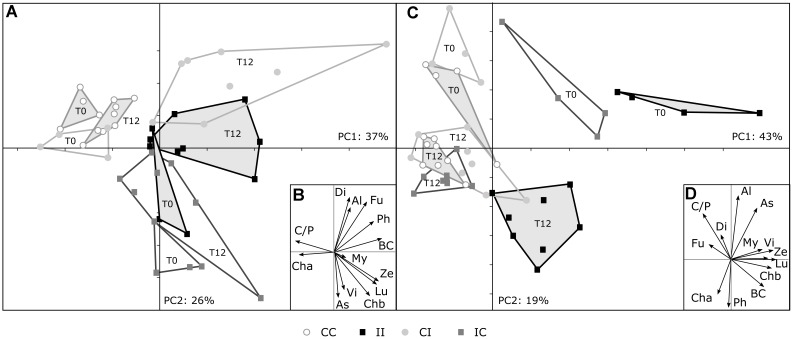
Principal component analysis on microbial photosynthetic communities from all the areas (CC, control – still control; II, impacted - still impacted; CI, control – then impacted; IC, impacted – then control) of *C. tagal* **(A)** and *R. mucronata* **(C)** mangrove zone, assessed using pigments as biomarkers. The inserts **(B,D)** give the factorial maps of the pigments. Samples correspond to the beginning (T0) and to the end (T12) of the experiment. As, astaxanthin; Al, alloxanthin; BC, β-carotene; Cha, chlorophyll *a*; Chb, chlorophyll *b*; C/P, chlorophyll a/pheophytin a; Di, diadinoxanthin; Fu, fucoxanthin; Lu, lutein; My, myxoxanthophyll; Ph, pheophorbide a; Vi, violoxanthin; Ze, zeaxanthin.

The structures of microbial photosynthetic communities were assessed using pigments as biomarkers. As illustrated by PCAs performed on all the areas at the beginning and at the end of the experiment (Figure 10), PW discharges strongly modified the structure of phototrophic communities. In C-CC area, the communities were associated with higher chlorophyll *a* concentrations and chlorophyll *a*/pheophytin *a* ratio, while in C-II area the communities were associated with higher proportions (ratio to chla concentration) of chlorophyll *b*, lutein, zeaxanthin, β-carotene, myxoxanthophyll, astaxanthin, and violaxanthin (Figure 10A). The latter pigments are indicators of the presence of green algae and cyanobacteria. At T0, communities from the C-CI and C-IC areas were quite similar to those of C-CC and C-II areas, respectively. After 12 months, the samples from C-CI are more distant from those of C-CC area and closer from those of C-II area. They were notably characterized by higher concentrations in fucoxanthin (diatoms). In the meantime, samples from C-IC area remained apart from samples from C-CC area, but moved away from C-II samples. In the *R. mucronata* mangrove zone, the impacted area was characterized by lower proportions of fucoxanthin (diatoms) and higher proportions of chlorophyll *b*, lutein, zeaxanthin, β-carotene and violaxanthin (Figure 10B). During the 12 months experiment, all the communities showed higher chlorophyll *a* content, indicating higher densities of phototrophic microorganisms. After 12 months, samples from R-CI area remained close from samples from R-CC area, showing no short-term impact on phototrophic communities. In contrast, samples from R-IC area came closer from samples from R-CC area, showing a good resilience of these communities.

**FIGURE 11:**
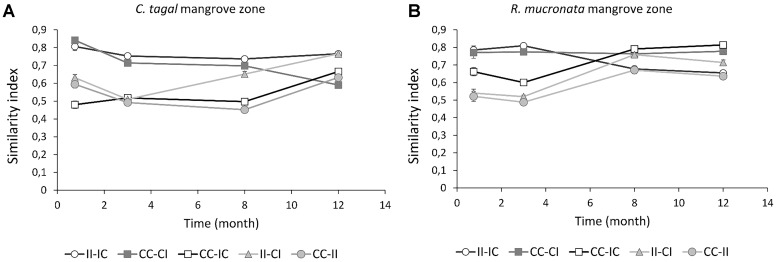
Dynamic of Bray-Curtis similarity index of microbial photosynthetic communities in sediments of *C. tagal* **(A)** and *R. mucronata* **(B)** mangrove zones. Each curve represents the similarity between two areas (mean ± SE, *n* = 16).

The structures of the phototrophic communities were also compared by using the Bray-Curtis similarity index. The similarity between communities from C-IC and C-II areas remained stable over time, while the similarity between communities from C-IC and C-CC areas followed the same dynamics than similarity between communities from C-II and C-CC areas, showing no or low resilience. In contrast, the similarity between communities from R-IC and R-CC areas significantly increased after 8 months without discharge, showing a resilience of these communities (Figure 11). This was associated with a significant decrease of similarity between communities from R-IC and R-II areas after 8 months. This is very consistent with the results of the PCA. A short-term impact was observed after 8 months in C-CI area as shown by the increase of the similarity between communities from C-CI and C-II areas, and the decrease of similarity between communities from C-CI and C-CC areas, which is consistent with the observations on the PCA. At the opposite, the similarity between communities from R-CI and R-CC areas did not change overtime therefore no short-term impact was observed in *R. mucronata* mangrove zone, as seen on the PCA. After 12 months of the present experiment, the similarities between control and long-term impacted (II) areas were not different in *R. mucronata* and *C. tagal* zones (Table 3), while the similarity between control and short-term impacted (CI) areas was significantly lower in the *C. tagal* zone. The structure of phototrophic communities in areas where PW discharges were stopped (IC) had a higher similarity with control communities (CC) and lower similarity with impacted communities (II) in the *R. mucronata* zone (Table 3).

**Table 3:** Mean similarity Bray-Curtis index between areas in the *C. tagal* and the *R. mucronata* areas (mean ± SE, *n* = 64).

	Long-term impact	Short-term impact	Resilience	
			
	CC-II	CC-CI	IC-CC	IC-II
*C. tagal*	0.63 ± 0.01	0.67 ± 0.02	0.67 ± 0.01	0.77 ± 0.01
*R. mucronata*	0.64 ± 0.01	0.77 ± 0.01**^∗∗∗^**	0.81 ± 0.0**^∗∗∗∗^**	0.65 ± 0.01**^∗∗∗^**


*Asterisks indicate significant differences with the respective control (^∗∗∗^*p* < 0.001).*

## Discussion

### Sediment and Porewater Composition

Although mangroves are rich in organic matter, they are deficient in nutrients (Boto and Wellington, 1984), particularly in nitrogen (Levy-Booth et al., 2014) and phosphorous (Prasad, 2012). PW discharges during more than 7 years induced an increase in C and N sediment content in both mangrove zones. These proportions remained stable in the impacted areas during the 12 months of the present study, indicating that sediment had reached an equilibrium state despite the continuous PW discharge (Figure 3). This suggests that additional C and N input in impacted areas were quickly removed from sediments either by microbial activities (Wu et al., 2008; Tam et al., 2009) and/or by vegetation assimilation (Ganguly et al., 2009; Lambs et al., 2011). This is confirmed by the absence of C:N ratio modification between T0 and T0 + 12 months in most areas except for the R-II and C-CC areas. In this study, the PW were mainly composed of ammonium which is the most abundant and the more plant-available form of nitrogen found in mangrove ecosystem (Purvaja et al., 2008). At long-term the ammonium concentration strongly increased in surface water in the impacted areas. This induced an increase of mangrove tree growth in impacted areas of both mangrove zones (4 fold in *C. tagal* area and 1.7 fold *R. mucronata* area) (Capdeville et al., 2018). Besides, microbial communities involved in N-cycle like aerobic nitrifying bacteria participate to the recycling of ammonium from PW by the formation of nitrate in the first layers of mangrove sediments (Alongi et al., 1992). Thus, nitrate can be up-taken by mangrove trees (Purvaja et al., 2008) or completely removed from sediments through the denitrification activity under gaseous forms (N_2_, NO, N_2_O) (Fernandes et al., 2012). Ammonium can also be removed from sediments *via* anammox by anammox bacteria which release N under gaseous form (N_2_) (Li and Gu, 2013). This metabolic process is rather difficult to measure. However, the level of anammox seems to be low in mangrove ecosystem, potentially due to low nitrite concentration (Fernandes et al., 2012) and moreover it decreases when temperature reach 37°C (Bertrand et al., 2011). Therefore, it seemed more relevant to focus on the processes of nitrification and denitrification.

At short term (12 months), a partial return to initial proportions on N- and C-proportions was observed, only in the *R. mucronata* mangrove. The N and C contents remained higher in R-IC area compared to control areas but they decreased compared to the R-II area (Figures 3E,F). This stability may partially be explained by the leaching of sediments during each tide, which is more marked in *R. mucronata* mangrove zone than in the *C. tagal* one. However, the volume of PW discharges has been chosen after determination of sediment porosity, taking into account crab hole volume and density, to avoid accumulation on the surface and direct leaching by the tides. Though the PW bring phosphorous and PO_4_^3-^ concentration significantly increased in porewater, the variations in sediment P content were low, with a tendency of decrease in impacted areas. P cannot be removed from sediment under gaseous form like N, but inorganic P can be up-taken by vegetation and organic P by microbial biomass. This decrease despite phosphate input may be explained by the higher biomass of trees in the impacted areas, where the vegetation growth significantly increased (Capdeville et al., 2018). In the same experimental system, Herteman (2010) showed an immobilization of P in sediment beyond 60 cm of depth after 18 months of discharges. It would be relevant to measure the P-proportion such deep layer in sediment after 9 years of discharges. Indication of porewater chemistry in the course of the experiment would have been useful to better understand the underlying mechanisms. Such analyses were planned but unfortunately canceled due to administrative issues.

### Total Density of Bacteria

Our results highlighted some positive effects of PW inputs on bacteria densities, in both mangrove zones (Figures 4B,D). However, if there were both short-term and long-term effects in the *C. tagal* zone with a 3–4 times increase, only a short-term effect was observable in the *R. mucronata* zone. The bacterial density remained high in the C-IC area, indicating the absence of resilience. As a whole, these results point to a higher stability of bacterial densities in the *R. mucronata* zone, as for the elementary composition of sediments. Contrasted effects have been observed in sediments of mangrove submitted to anthropogenic inputs or N inputs, with either marginal effects on bacterial densities (Fernandes et al., 2014), negative effects (Luo et al., 2017) or positive effects (Tam et al., 2009; Wickramasinghe et al., 2009; Bouchez et al., 2013). This highlights the wide range of potential response of bacterial densities in mangrove sediments. It is known that mangrove microbial communities are strongly influenced by the environmental parameters like temperature, humidity, pH (Alongi et al., 1992), salinity (Tam, 1998), C:N ratio, dissolved oxygen and nutrient concentrations (Li and Gu, 2013). Besides, the presence of aerobic and anaerobic bacteria depends on the oxygen level in sediments which is modulated by the tides, the bioturbation activity, and the presence of mangrove tree roots (Purvaja et al., 2008). These biotic and abiotic parameters can all modify the abundance of bacteria in mangrove sediments (Chakraborty et al., 2015). In the studied experimental system, PW discharges induced modifications in all these parameters, strongly impacting the environmental conditions. The effects of wastewaters can therefore be both direct and indirect, *via* these modifications of the environmental parameters.

### Abundances and Activity of Microorganisms Involved in N-Cycle

In this study, our goal was to assess functional resistance and resilience of the microbes involved in N-cycle. Although temporal dynamics of community structure may give some mechanistic details of how the communities responded to treatments, the acknowledged redundancy of these functions among many microbial groups restrained us from compiling these data. We chose instead to focus on their relative abundances (by qPCR) and measure of potential activities that are more integrative values of functional potential of the different groups. Within bacterial communities, the densities of some specific groups were differently regulated in response to the treatments. At long term, PW discharges induced an increase in the abundances of denitrifying bacteria harboring the gene *nos*Z clade I in both mangrove zones. At short term, the increase in density was significant only in the *C. tagal* mangrove. Similar results were observed in other mangroves impacted by the wastewaters (Tam, 1998; Tam et al., 2009; Wickramasinghe et al., 2009; Bouchez et al., 2013; Huang et al., 2017). The N-inputs in mangrove sediments induced an increase of abundance of bacteria involved in N-elimination. This led to a stimulation of the potential denitrification activity in impacted areas of both mangrove zones, as it was demonstrated by Kristensen et al. (1998). Consistently with the results obtained for the abundance of *nos*Z clade I gene copies, the short-term impact on denitrification activity was revealed after 8 months in both mangrove zones and was faster in *C. tagal* mangrove zone than in *R. mucronata* mangrove zone. In a mangrove exposed to shrimp effluents, all N-cycling processes were stimulated between 2 and 12-fold (Molnar et al., 2013). The denitrification can be also favored by the bioturbation activity of crab present in these areas (Capdeville et al., 2018). Indeed the crab promote the entry of oxygen in depth which stimulates the production of nitrate *via* the nitrification and favor the transformation of nitrate into N_2_ *via* the denitrification during anoxia period (Kristensen et al., 1998). In terms of resilience, a clear effect of the interruption of PW discharge was observed on the potential DEA while no clear trends could be observed on the abundance of denitrifying bacteria. It is possible that these bacteria are still present in the sediment but are less active. An analysis of *nos*Z clade I gene transcript with RT-QPCR might have explained these results. Here again, for the abundance and activity of denitrifying bacteria, the sediment from the *R. mucronata* zone appear more resistant and more resilient.

The presence of nitrifying communities in sediments could explain the N-elimination from sediments in impacted areas thanks to the coupling of nitrification and denitrification. However, these two processes were not directly correlated when considering the two zones or treatments separately (data not shown). The community of nitrifiers was dominated by the archaea (AOA) rather than by the bacteria (AOB), as described in marine ecosystems by Nicol and Schleper (2006) or soils with low pH, low organic matter content or low NH4+ concentrations (Bates et al., 2011; Assemien et al., 2017) or these two groups of microorganisms, the variations in abundance of both *amo*A genes overtime in all areas of the two mangrove zones, showed no clear patterns of response to the treatments. Cao et al. (2011) revealed the presence of AOA and AOB in polluted mangrove sediments, with higher abundance of the AOA but higher diversity of the AOB. According to these authors, the abundances of AOA and AOB were correlated with the pH and the temperature while the AOA:AOB ratio was correlated with the ammonium concentration (Cao et al., 2011). However, in the present study, in impacted areas submitted to high concentrations of ammonium the AOA:AOB ratio was not higher than in control areas. At short term, only the AOA abundance increased, in both mangrove zones, and at long term the effects of PW discharged were mainly visible on the AOA abundances. Although the AOB seem at first to be less sensitive than AOA to the anthropic disturbance applied in the study, we found that the relative abundance of AOB, but not AOA, was correlated with NEA in response to our treatments (Figure 8). Although no significant correlation was observed in the control treatments, AOB copy numbers were negatively correlated to NEA in the two impacted treatments (I-I, and C-I), suggesting the activity and the relative abundances of this group were affected by PW inputs. This trend was found for both *R. mucronata* and *C. tagal* and was more marked for I-I than C-I. More surprisingly, in the I-C treatment, this negative correlation was released, suggesting a quick resilience of the NEA and associated AOB population. A partial resilience for the abundance of AOA was also visible only in the *R. mucronata* mangrove zone. In the literature, the nitrifying communities of mangrove sediment were also stimulated by the wastewaters (Tam, 1998; Tam et al., 2009; Wickramasinghe et al., 2009; Bouchez et al., 2013) without any effect on the structure of the nitrifiers (Tam et al., 2009; Wickramasinghe et al., 2009). Similarly, an amendment of ammonium on mangrove sediment stimulated the growth of AOA and AOB whereas an amendment of nitrite inhibited them (Li and Gu, 2013). However, both amendments altered the composition of AOA and AOB. It would be relevant to study the composition of AOA and AOB in presence of wastewaters in the mangrove areas of the present study. The nitrification process occurs at the surface of sediment or in micro-oxic zones generated by the mangrove tree roots and the sediment turnover carry out by the bioturbation of crab (Purvaja et al., 2008). It should be noted that we used laboratory methods to measure potential nitrification and denitrification activities. This gives reliable indications on the ability of microorganisms to nitrify or denitrify in standard conditions, but *in situ* effective activities may be slightly different.

### Structure of Microbial Photosynthetic Communities

Our results, using pigment content as a biomarker of taxonomic composition, indicate a significant long-term impact of PW discharges on the structure of phototrophic microorganism communities in both mangrove zones. Several studies demonstrated that the phototrophs like the green algae, diatoms and cyanobacteria were stimulated in mangrove sediment exposed to wastewaters (Tam et al., 2009; Wickramasinghe et al., 2009; Bouchez et al., 2013). Here no long-term effect of PW was observed on the quantity of chlorophyll *a*, a proxy of the density of phototrophic microorganisms. The decaying leaves may contribute to the detected chlorophyll *a* in sediment surface, but this interference is certainly low because the litter is rapidly trapped, buried and consumed by the crabs feeding on leaves (Camilleri, 1992; Botto et al., 2006).

As for heterotrophic microorganisms, the phototrophs can be impacted directly by the PW but also indirectly by the modification of their environment. Indeed, the PW induced a strong growth of mangrove trees resulting in a canopy closure and therefore a decrease of light intensity at the sediment surface (Capdeville et al., 2018). This decrease of light can strongly affect the phototrophs. The results highlighted an increase of green algae and cyanobacteria, which are often found in eutrophic ecosystems. In R-II, this was associated with a lower number of diatoms, as suggested by the negative correlation with fucoxanthin (Figure 10). These modifications may be critical for the functioning of the ecosystem. Indeed, phototrophic microorganisms are an essential food source for many organisms, like the meiofauna. Changes in the available food may trigger modifications of the meiofauna community. Among meio-organisms, the nematodes, which are abundant in mangrove sediment (Capdeville et al., 2018), participate to all the pathways of energy transfer of microbial carbon to higher trophic levels in benthic food webs and are essential for the functioning of benthic ecosystems (Schmid-Araya and Schmid, 2000; Carpentier et al., 2014). Bottom–up impacts on these bioturbation organisms may also degrade the oxygenation of sediment.

Though at long-term the impact seems equivalent in both mangrove zones, at short term it was lower in the *R. mucronata* zone while the resilience was more marked in this zone (Table 3).

## Conclusion

At long-term, the anthropic disturbance – daily discharges of PW – resulted in higher densities of total bacteria, denitrifying bacteria, and in a lower extent AOA. This was associated with an increase in the denitrification activity and modifications of the communities of photosynthetic microorganisms. The short-term responses of microbial communities from *C. tagal* and *R. mucronata* mangrove zones, as well as their potential resilience strongly depended on the mangrove zone. Microorganisms from the *R. mucronata* mangrove zone were more resistant and resilient than the one from the *C. tagal* zone, at least for the considered parameters. This confirms our hypothesis. Despite the fact that only two zones were tested, the stability of microbial communities clearly varies along the environmental gradients that structure the mangrove ecosystem. The higher stability of the *R. mucronata* zone may be explained by the local adaptation of the microorganisms to anoxia (occurring during high tides, likely increased by nutrient inputs) but also to high vegetation cover. Indeed, the PW discharges induced a stronger development of the vegetation (Capdeville et al., 2018). Since in our study site the *C. tagal* trees have a much weaker development than the *R. mucronata* trees, the contrast between impacted and control areas was much higher in the *C. tagal* zone. This is consistent with our hypothesis stating that the stability of communities should be higher when the disturbance exacerbates natural constraints. Concerning heterotrophic microorganisms, information on the abundance of DNA transcripts would have been relevant, but they were not available due to technical issues. Indeed, qPCR help to quantify the cells that are or were present at a given time, but give no information about the proportion of active cells. Information on the composition of communities, through NGS methods, would also bring complementary information, though the focus of the study was the resistance and resilience of the functions associated with the communities.

Although we only considered the microorganisms in this study, our results emphasize the heterogeneity of mangrove stability. Another spatial constraint is the extent of the disturbance. As for bigger organisms, the resilience of microorganisms is enhanced by connectivity with undisturbed communities representing spatial refuges (Baho et al., 2012). These points should be carefully taken into account in both conservation policy and mangrove exploitation, to favor higher recovery. Moreover, we confirm here the high potential of using mangroves for bioepuration of PW.

The short-term impact and the resilience were overall observed first on the denitrification activity (after 8 months) and second on the microbial abundances (after 12 months). Although the microbial structure may have been strongly modified, the microorganisms maintained the microbial functions in mangrove sediment thanks to their functional redundancy. The resilience observed is thus associated to a functional recovery. Besides, the microbial activity and the vegetation uptake seem to remove efficiently the C, N, and P nutrients from wastewaters accumulated in sediments. The equilibrium state was faster to reach in *R. mucronata* mangrove zone. This confirms the strong potential of the use of *R. mucronata* mangrove zone in bioepuration of PW. To our knowledge, this is the first time that the resilience capacity of microbial community was studied *in situ* in two mangrove zones after a long-term exposure to PW discharges.

## Author Contributions

FF, J-LR, JL, and TP designed the experiments. CC, FF, J-LR, TP, and JL contributed to the field work. CC, JL, and JG made the laboratory analyses. The manuscript was first written by CC, and then improved by the other authors.

## Conflict of Interest Statement

The authors declare that the research was conducted in the absence of any commercial or financial relationships that could be construed as a potential conflict of interest.
